# Biocontrol efficacy of *Trichoderma asperellum* TA-1 against *Rhizoctonia solani* AG-3 and its underlying mechanisms

**DOI:** 10.3389/fmicb.2026.1899737

**Published:** 2026-10-13

**Authors:** Suoni Liu, Yonggang Xia, Can Wang, Bin Hu, Cixi Zhang, Bing Wang, Dong Li, Tianbo Liu, Qianjun Tang

**Affiliations:** 1 College of Plant Protection, Hunan Agricultural University, Changsha, Hunan, China; 2 Hunan Academy of Forestry, Changsha, Hunan, China; 3 Hunan Provincial Branch, China National Tobacco Corporation, Changsha, Hunan, China; 4 Shaoyang Branch, Hunan Provincial Company of China National Tobacco Corporation, Shaoyang, Hunan, China

**Keywords:** biological control, *Rhizoctonia solani*, spore suspension, tobacco, *Trichoderma asperellum*

## Abstract

**Introduction:**

*Rhizoctonia solani* Kühn is a globally distributed soilborne pathogen responsible for severe diseases in various crops, including rice, wheat, tobacco, and maize, causing substantial economic losses. This study characterized a *Trichoderma* strain, TA-1, with significant biocontrol efficacy against tobacco target spot disease.

**Methods:**

TA-1 was isolated and identified through morphological characterization and molecular analysis as *Trichoderma asperellum*. Qualitative assays were conducted to assess ammonia production and laccase, amylase, and protease activities. Solid-state fermentation trials were performed to optimize spore production conditions, and greenhouse assays were conducted to evaluate biocontrol efficacy. Microscopy, scanning electron microscopy, and transcriptomic analyses were used to characterize the interaction between TA-1 and *R. solani* AG-3.

**Results:**

Qualitative assays indicated ammonia production and laccase, amylase, and protease activities in TA-1. The optimal spore production conditions were wheat bran substrate, 0.5% glucose, 1.5% ammonium sulfate, a water-to-substrate ratio of 1:1, and a fermentation duration of 6 days, under which the spore yield reached 4.74 × 10^9^ CFU/g dry substrate. Greenhouse assays showed that both TA-1 spore suspension and fermentation filtrate significantly suppressed disease symptoms caused by *R. solani* AG-3, with the spore suspension exhibiting stronger inhibition. Microscopy and scanning electron microscopy revealed clear mycoparasitic features during the TA-1–AG-3 interaction, including hyphal attachment and coiling, structural damage to pathogen hyphae, and colonization of the sclerotial surface. Transcriptomic analysis further identified strong upregulation of *TrAFT101_005492*, encoding a CFEM domain protein (log_2_FC = 4.99), and *TrAFT101_000974*, encoding a lipoxygenase (log_2_FC = 4.73), suggesting their potential association with the mycoparasitic interaction.

**Discussion:**

Overall, *T. asperellum* TA-1 possesses broad antagonistic activity and considerable biocontrol potential, supporting its further evaluation as a biological control agent for sustainable disease management.

## 1 Introduction

*Rhizoctonia solani* Kühn is a globally distributed soil-borne pathogenic fungus with broad host adaptability and strong environmental resilience (Gonzalez et al., 2011). This pathogen is capable of causing a wide range of devastating crop diseases, including rice sheath blight, potato black scurf, maize banded leaf and sheath blight, and tobacco target spot disease (Zheng et al., 2013). Under unfavorable environmental conditions, *R. solani* produces sclerotia–dormant structures composed of compact mycelia–which enable long-term survival in soil. When environmental conditions such as temperature and humidity become favorable, sclerotia germinate to produce infectious hyphae, which penetrate host tissues by secreting a suite of cell wall-degrading enzymes, such as cellulases and pectinases, and by releasing pathogenicity-associated toxins (Duggar, 1915). In this way, *R. solani* successfully invades host plants and poses a serious threat to crop production security.

Based on hyphal anastomosis compatibility, *R. solani* has been classified into 14 Anastomosis Groups (AGs), designated AG-1 to AG-13 and AG-BI (Xu et al., 2024). These AGs differ markedly in morphology, host range, virulence, sclerotial development, toxin production, and cell wall-degrading enzyme profiles. AG-1 is the predominant pathogenic type causing rice sheath blight and typically produces water-soaked lesions that expand along leaf sheaths and blades (Savary, 1995; Ren et al., 2017). In contrast, tobacco target spot disease is primarily associated with AG-3, AG-2-1, and AG-5, which produce characteristic concentric ring lesions on leaves. Lesion expansion can lead to central necrosis and tissue abscission, reducing leaf quality and yield (Gonzalez et al., 2011). These AG-dependent differences in host specialization and pathogenic behavior provide an important theoretical foundation for precision disease control.

Traditionally, the management of *R. solani* diseases has relied on cultural practices, crop rotation, residue management, resistant cultivars where available, and chemical fungicides. However, repeated fungicide use can contribute to environmental contamination, soil microbiome disturbance, and selection for fungicide-resistant pathogen populations. Consequently, biological control has attracted increasing attention in integrated disease management programs due to its environmental compatibility and sustainability. The management strategy for *R. solani* diseases is gradually shifting toward biocontrol approaches, with fungi of the genus *Trichoderma* emerging as a major focus due to their multifaceted mechanisms of action. *Trichoderma* spp. can exert direct antagonistic effects, including mycoparasitism (e.g., *T. asperellum* causing mycelial deformation in *Fusarium oxysporum*, *T. koningiopsis* lysing *R. solani* hyphae), nutrient competition, and secretion of antifungal metabolites such as peptaibols and other antibiotics (Shaw et al., 2016; Díaz-Gutiérrez et al., 2021). They can also act indirectly by inducing systemic resistance and enhancing rhizosphere microbial balance, thereby improving plant defense capacity (Tyśkiewicz et al., 2022). These direct and indirect mechanisms have been reported across *in vitro* confrontation assays, greenhouse studies, and soil- or plant-based disease suppression systems (Tyśkiewicz et al., 2022; Arasu et al., 2023).

Notably, *Trichoderma* spp. possess significant plant growth-promoting capabilities, benefitting diverse crop families such as Fabaceae (e.g., *Vigna mungo*), Solanaceae (e.g., tobacco), Poaceae (e.g., maize), and Brassicaceae (e.g., cabbage). For example, *T. gamsii* has been reported to stimulate plant growth and enhance *V. mungo* resistance to root rot through lytic activity (Arasu et al., 2023). An endophytic *Trichoderma longibrachiatum* strain WKA55 promoted peanut germination and suppressed mycotoxigenic fungi (Al-Askar et al., 2022), while *T. harzianum* inoculation reduced clubroot disease in Chinese cabbage by regulating the rhizosphere microbial community (Li et al., 2020). These findings demonstrate that *Trichoderma* spp. can function as ideal microbial inoculants that integrate biocontrol efficacy with growth promotion, simultaneously suppressing pathogens and enhancing crop yield and quality.

Progress has also been made in the biocontrol of *R. solani*. For example, *Trichoderma pubescens* has been reported to induce systemic resistance in tomato challenged by *R. solani* (Behiry et al., 2023), and recent molecular studies have identified *Trichoderma* genes involved in recognition, mycoparasitism, nutrient competition, and defense activation against *R. solani* (Abbas et al., 2022). However, research on biocontrol targeting tobacco target spot disease caused by different *R. solani* AGs, particularly AG-3 and AG-2-1, remains limited. In the present study, a *Trichoderma* strain TA-1 was isolated from paddy–tobacco rotation fields in Hunan Province, China, and identified as *T. asperellum* based on morphological and molecular characteristics. TA-1 exhibited remarkable control efficacy against major local diseases, including rice sheath blight and tobacco target spot. Multi-dimensional analyses revealed that TA-1 not only antagonizes *R. solani* but also promotes the growth of both rice and tobacco. Furthermore, transcriptomic analysis was used to connect observable antagonistic phenotypes with candidate molecular pathways during the TA-1 and *R. solani* AG-3 interaction, providing a theoretical basis for the development of broad-spectrum biocontrol agents.

## 2 Materials and methods

### 2.1 Isolation, purification, and identification of the target strain

Rhizosphere soil samples were collected from diseased plots in a tobacco–rice rotation area in Chenzhou, Hunan Province, China. Under aseptic conditions in a laminar flow cabinet, the soil samples were thoroughly mixed, and 10g was transferred into a 250mL Erlenmeyer flask containing 90mL of sterile water and sterile glass beads. The flask was shaken on a rotary shaker for 30 min to evenly disperse the soil in the diluent, resulting in a 10^−1^ soil suspension. A tenfold serial dilution was carried out to obtain a 10^−4^ suspension. Sterile PDA (potato dextrose agar) plates were prepared, cooled, and inoculated with 10^−4^ soil suspension for fungal colony observation. Plates were incubated in an inverted position at 28°C for 5–7 days. Colonies with distinct morphologies were selected and subjected to 3–4 rounds of sub-culturing to obtain pure isolates, which were stored at 4°C.

The purified strain TA-1 was inoculated at the center of a 90mm PDA plate and incubated at 28°C for 3 days to examine colony morphology and growth rate. Preliminary morphological identification was performed following standard fungal identification references (Lieckfeldt et al., 1999). After 3 days, colonies were covered with white flocculent mycelia, and after 5 days, green conidia appeared, with color intensity and quantity increasing over time until the colony covered the entire plate. Fresh mycelia were scraped for molecular identification. DNA was extracted using a CTAB-based fungal DNA extraction protocol (Dar et al., 2025), and PCR amplification was performed with three pairs of primers (ITS1/ITS4, CAL-228F/CAL-737R, and EF1-728F/EF1-986R; detailed in Supplementary Table 1). The 25 μL PCR reaction mixture contained: ddH2O (10.5 μL), PCR mix (12.5 μL), forward primer (0.5 μL), reverse primer (0.5 μL), and DNA template (1.0 μL).

The PCR program was as follows: initial denaturation at 94°C for 5 min; denaturation at 94°C for 30 s; annealing at 55°C (ITS) or 52°C (CAL and EF1) for 45 s; extension at 72°C for 1 min; final extension at 72°C for 10 min; hold at 4°C. PCR products were subjected to bidirectional sequencing. The resulting sequences were compared against the NCBI database using the BLAST program for nucleotide sequence homology search. A phylogenetic tree was constructed using MEGA 10.0 software to determine the taxonomic position of TA-1.

### 2.2 Qualitative functional trait screening of TA-1

These assays were performed as qualitative screening tests to determine the presence of selected functional traits in TA-1 and were not intended for quantitative enzyme activity measurements.

#### 2.2.1 Ammonia production assay

A 5 mm mycelial plug was excised from the margin of a 7-day-old TA-1 colony grown on PDA and inoculated into liquid broth containing 4% peptone. The culture was incubated statically at 28°C for 7 days. After incubation, the culture was centrifuged at 8,000rpm for 5 min, and 1.0 mL of supernatant was mixed with 100 μL of Nessler’s reagent. Ammonia production was determined by observing the colorimetric change of the solution after adding Nessler’s reagent. A yellow to brown reaction was interpreted as positive ammonia production (Nautiyal, 1999).

#### 2.2.2 Laccase activity assay

A total of 300mL of PDA medium was melted and cooled to approximately 55°C before adding guaiacol (120 μL). The mixture was poured into Petri dishes. A 5mm mycelial plug of TA-1 was placed at the center of each guaiacol-supplemented PDA plate and incubated at 28°C. Laccase activity was indicated by the appearance of brown or dark brown zones around the colony (Lakshmanan and Sadasivan, 2016).

#### 2.2.3 Protease activity assay

Protease detection medium was prepared with 1% agar and 5% skim milk powder, autoclaved at 115°C for 30 min, and poured into plates. TA-1 mycelial plugs were inoculated onto the plate center and incubated at 28°C. The appearance of a clear zone surrounding the colony indicated protease activity (Wu et al., 2017).

#### 2.2.4 Amylase activity assay

Amylase detection medium was prepared with the following composition (g/L): soluble starch, 20.0; KCl, 0.5; NaNO_3_, 2.0; K_2_PO_4_, 1.0; MgSO_4_⋅7H_2_O, 0.5; NaCl, 5.0; agar, 18.0, and distilled water to 1L. The medium was autoclaved at 115°C for 30 min and poured into plates. TA-1 mycelial plugs were inoculated centrally and incubated at 28°C. After sufficient mycelial growth, iodine solution was added around the colony margin. Disappearance of blue coloration or the appearance of a clear zone indicated amylase activity (Wang et al., 2021).

### 2.3 Antagonistic activity of *T. asperellum* TA-1 against plant pathogenic fungi

To establish the broad-spectrum biocontrol baseline of strain TA-1 before focusing entirely on our primary target, *R. solani* AG-3, a dual culture assay was used to evaluate the antagonistic activity of *T. asperellum* TA-1 against seven phytopathogenic fungi: *Phyllosticta musarum* (banana freckle pathogen), *Phytophthora fragariae* (strawberry red core pathogen), *Fusarium commune* (tobacco root rot pathogen), *Piricularia oryzae* (rice blast fungus), *Alternaria alternata* (tobacco brown spot pathogen), *Sclerotium rolfsii* Sacc (pepper southern blight pathogen), and *R. solani* AG-3 (tobacco target spot pathogen). All strains were preserved at the Plant Pathology Laboratory, Hunan Agricultural University.

Mycelial plugs (5mm in diameter) were cut from the actively growing margins of pathogen cultures and placed 3cm from the edge on one side of a PDA plate. An equal-sized plug of TA-1 was placed on the opposite side of the plate, also 3cm from the edge. For the control (CK), only the pathogen was inoculated. Plates were incubated at 28°C for 72h, after which the radial growth of the pathogen toward the TA-1 colony was measured.

The percentage inhibition of radial growth (PIRG) was calculated according to a published radial-growth inhibition method (Darlis et al., 2023) as:


PIRG(%)=[(R1-R2)/R1]×100

where

R1: Radial growth of plant pathogenic fungi in control plates.

R2: Radial growth of plant pathogenic fungi in the presence of antagonist (*T. asperellum* TA-1).

### 2.4 Greenhouse evaluation of TA-1 for the control of tobacco target spot caused by AG-3

Healthy tobacco and rice plants, uniform in growth and morphology, were used for the greenhouse bioassay. For tobacco, a modified needle puncture inoculation method was applied: mycelial plugs (5mm diameter) of *R. solani* AG-3 from the actively growing margin were placed mycelium-side down onto puncture wounds made approximately 3cm from the midrib on either side of the leaf using a sterile needle.

Four treatments were applied:

- A (positive control): inoculated with AG-3 mycelial plugs and sprayed with 20mL sterile water;
- B: inoculated with AG-3 mycelial plugs and sprayed with 20 mL of 10-fold diluted sterile TA-1 fermentation filtrate (v/v);
- C: inoculated with AG-3 mycelial plugs and sprayed with 20 mL of TA-1 spore suspension (1.00 × 10^7^ CFU/mL);
- D (negative control): not inoculated with AG-3, but sprayed with sterile water.

The fermentation filtrate was obtained by removing fungal biomass from TA-1 liquid culture by centrifugation and filtration through a sterile membrane before dilution with sterile water. The spore suspension was prepared from freshly sporulating TA-1 cultures and adjusted to 1.00 × 10^7^ CFU/mL before spraying. Following treatment, the inoculation sites were misted every 2 h with sterile water to maintain moisture. Plants were maintained in a greenhouse at 28°C. Each treatment included three biological replicates, each replicate consisting of three plants, with two functional leaves inoculated per plant.

Three days after inoculation, the vertical and horizontal lesion diameters were measured with a vernier caliper, and their mean was used as the lesion diameter. Disease control efficacy (DCE) was calculated as DCE (%) = [(Lcontrol - Ltreatment)/Lcontrol] × 100, where Lcontrol and Ltreatment are the mean lesion diameters of the pathogen control and the TA-1 treatment, respectively. Lesions were also photographed for disease assessment.

### 2.5 Observation of mycoparasitism of TA-1 against AG-3

To investigate the mycoparasitic mechanism of TA-1 against AG-3, TA-1 was co-cultured with AG-3 and another pathogen on PDA plates. When the pathogen colonies came into contact with TA-1 colonies, agar blocks (∼1cm on each side) containing mycelia from both sides of the interaction zone were excised for optical microscopy (OM) and scanning electron microscopy (SEM) analysis.

For SEM sample preparation, agar blocks were fixed in 2.5% glutaraldehyde at room temperature for 2h, dehydrated sequentially in graded ethanol and acetone series (30%, 50%, 70%, 90%, 100%, each for 10–15 min), and dried in a critical point dryer (Quorum K850). Samples were sputter-coated with gold using an ion sputter coater (HITACHI MC1000) and examined under a scanning electron microscope (HITACHI Regulus 8100) to observe the morphological interactions between TA-1 hyphae and pathogen hyphae.

### 2.6 Qualitative observation of sclerotial degradation of AG-3 by TA-1

To examine the colonization of AG-3 sclerotia by TA-1, AG-3 was cultured on PDA plates at 28°C for 5–7 days to induce sclerotia formation. Sclerotia were surface-sterilized by immersion in 75% ethanol for 30 s, rinsed 5–7 times with sterile water, and placed on double-layer sterile lens paper in a 90 mm sterile Petri dish.

Freshly prepared TA-1 spore suspension (1.00 × 10^7^ CFU/mL) was applied to each treatment sclerotium at 40 μL, while the control group received 40 μL double-distilled water per sclerotium. This concentration matched the effective greenhouse spore-suspension treatment and provided a standardized inoculum density for direct sclerotial exposure. Each treatment had three replicates and was incubated at 28°C for 72 h. The sclerotia were then photographed and evaluated visually for TA-1 attachment and surface colonization. This assay was used for qualitative observation of TA-1 colonization on AG-3 sclerotia.

### 2.7 Methodology for optimizing solid-state fermentation of TA-1

To determine the optimal conditions for solid-state fermentation of TA-1, a series of systematic experiments were conducted. Initially, five candidate substrates–rice straw, corn stover, wheat bran, rice bran, and soybean meal–were assessed. Each substrate (10 g) was placed in a sterilized culture flask and combined with 10 mL of 1% glucose solution, followed by high-temperature sterilization. Subsequently, 1 mL of TA-1 spore suspension (1.00 × 10^7^ CFU/mL) was added to each flask. The flasks were incubated at 28°C under continuous light for 7 days.

After incubation, 1 g of each substrate was transferred to a 250 mL Erlenmeyer flask containing 100 mL of double-distilled water and shaken at 170 rpm for 1 h to ensure complete suspension of the TA-1 spores. Spore counts were determined using a hemocytometer to identify the optimal substrate, with each treatment performed in triplicate.

Following the identification of the optimal substrate, further experiments were conducted to screen for the best carbon and nitrogen sources. The selected carbon sources included fructose, sucrose, maltose, glucose, and starch, while the nitrogen sources included yeast extract, peptone, ammonium sulfate, urea, and potassium nitrate. Each culture flask contained 10 g of the previously determined fermentation substrate and 10 mL of distilled water, along with 0.1 g of the chosen carbon or nitrogen source. Each treatment was replicated in triplicate, and the incubation and counting methods were consistent with those described previously.

Based on the outcomes of the single-factor experiments, a three-factor, three-level orthogonal experiment was designed, resulting in a total of 12 unique combinations (without repetitions), each conducted in triplicate. The design scheme for this orthogonal experiment is presented in the accompanying table.

Finally, using the optimal fermentation parameters derived from the orthogonal experiment, a series of trials were performed to explore the optimal fermentation time for TA-1, with durations set at 2, 3, 4, 5, 6, 7, 8, 9, and 10 days. Day 0 and Day 1 data points were not included in the analysis as this period corresponds to the initial mycelial growth phase, during which sporulation is not yet detectable. Solid-state fermentation of TA-1 was executed at a constant temperature of 28°C for each specified duration, with each treatment replicated three times. Spore counting was performed using established protocols.

### 2.8 Transcriptomic analysis of TA-1 interaction with AG-3

To analyze transcriptional regulation during the interaction of *T. asperellum* TA-1 with *R. solani* AG-3, sterile cellophane discs were cut to fit the PDA plate surface. Fresh cultures of TA-1 and AG-3 were punched with a 5mm sterile cork borer, and mycelial plugs were placed 3 cm apart on opposite sides of the plate (AG-3 on one side, TA-1 on the other). Plates were incubated at 28°C for 48h until hyphae made contact. Mycelia (∼5mm on each side of the interaction zone) were scraped, frozen in liquid nitrogen for 30 min, and stored at −80°C until RNA extraction.

Three experimental groups were included: TA-1 cultured alone, *R. solani* AG-3 cultured alone, and mycelia collected from the interaction zone between TA-1 and *R. solani* AG-3 during dual culture. Each group consisted of three independent biological replicates, resulting in a total of nine RNA-seq samples. Total RNA was extracted, and raw reads were processed as follows: barcode sequences were separated using Illumina pipeline software (CASAVA v1.8.2), adapter sequences removed using Cutadapt (v1.4.1), and low-quality reads (average QPhred < 20, length < 50 bp) filtered using Trimmomatic (v0.39). Clean reads were aligned to the *T. asperellum* FT101 reference genome (assembly ASM2064786v1; GenBank accession GCA_020647865.1) using TopHat2 (v2.1.0). Gene expression levels were quantified as fragments per kilobase of transcript per million mapped reads (FPKM), with FPKM ≥ 1 considered indicative of gene expression.

Differentially expressed genes (DEGs) were identified using DESeq2 (v1.20.0) with padj < 0.05 and | log_2_FC| > 1. Functional annotation of DEGs was performed using Blast2GO (v2.5) against the NCBI non-redundant protein database (NR), followed by GO classification. GO and KEGG pathway enrichment analyses were performed using ClusterProfiler (v4.0).

To validate transcriptomic results, three up-regulated genes (*TrAFT101_005492*, *TrAFT101_000974*, *TrAFT101_009896*) and three down-regulated genes (*TrAFT101_003866*, *TrAFT101_004906*, *TrAFT101_003036*) were selected for RT-qPCR. RNA quality and concentration were assessed prior to reverse transcription using a TransScript One Step gDNA Removal and cDNA Synthesis SuperMix kit. Actin was used as the internal reference gene for RT-qPCR normalization (Luo et al., 2019). The RT-qPCR assay was used to verify the directional consistency of selected DEG expression patterns identified by RNA-seq; we acknowledge that future validation using an additional stable reference gene, such as Tubulin or EF-1α, would further strengthen normalization under mycelial interaction conditions. Primers were synthesized by Sangon Biotech (Shanghai) Co., Ltd., (sequences listed in Supplementary Table 2). qPCR was performed using Vazyme ChamQ Universal SYBR qPCR Master Mix on a QuantStudio 1 Plus Real-Time PCR system. Relative gene expression levels were calculated using the 2−ΔΔCT method.

### 2.9 Statistical analysis

All quantitative experiments included three independent biological replicates unless otherwise stated. Data are presented as mean ± standard deviation (SD). Statistical analyses were performed using SPSS 26.0 software. Differences among treatments were evaluated by one-way analysis of variance followed by Tukey’s honestly significant difference test for multiple comparisons. *P* < 0.05 was considered statistically significant.

## 3 Results

### 3.1 Isolation and identification of the antagonistic strain

The isolated strain, designated TA-1, was identified based on its morphological characteristics and multi-locus molecular analysis. When cultured on PDA, the colony of strain TA-1 initially exhibited white, aerial mycelia. As the culture aged, conidiation began, with the colony color progressively changing from light green to dark green until a dense layer of dark green spores covered the entire plate surface (Figures 1A, B). Under light microscopy, the hyphae of TA-1 were observed to be smooth and branched. The conidiophores were slender and typically branched, giving rise to phialides. The conidia were predominantly spherical and dark green in color (Figures 1C, D). For molecular identification, the internal transcribed spacer (ITS), calmodulin (CAL), and translation elongation factor 1-alpha (EF-1α) gene regions were amplified and sequenced. A multi-locus phylogenetic analysis was performed using ITS, CAL, and EF-1α sequences of TA-1 and representative *Trichoderma* reference strains. The analysis included *Trichoderma viride* TSL7a (Acc. No. PP101321.1), *T. rogersonii* GJS 04-158 (Acc. No. DQ370460.1), and *T. tomentosum* DAOM 195050 (Acc. No. AY605761.1) as outgroup taxa (Figure 1E).

**FIGURE 1:**
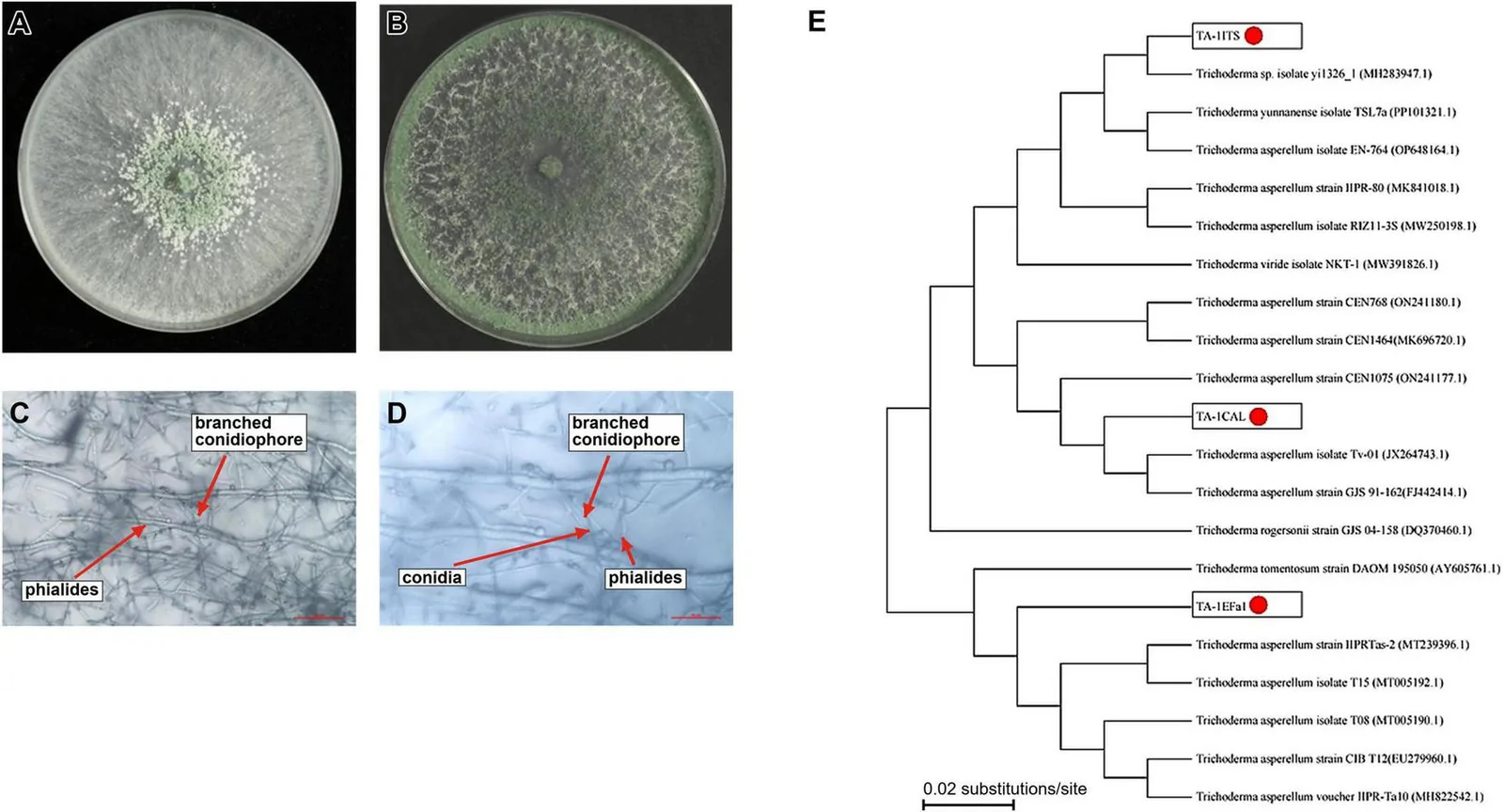
Morphological and phylogenetic identification of the antagonistic strain TA-1. **(A,B)** Colony morphology of *T. asperellum* TA-1 grown on PDA medium, initially producing white mycelia and gradually developing light green to dark green conidia until the entire plate was covered with dense dark green spores. **(C,D)** Optical micrographs of TA-1 showing smooth, branched hyphae, slender branched conidiophores, phialides, and mostly spherical conidia. **(D)** Shows a higher-magnification view of the selected diagnostic region in panel **(C)**; arrows indicate the key micromorphological structures. **(E)** Phylogenetic tree constructed using ITS, CAL, and EF-1α sequences of TA-1 and related *Trichoderma species*, with *T. viride* TSL7a (accession no. PP101321.1), *T. rogersonii* GJS 04-158 (accession no. DQ370460.1), and *T. tomentosum* DAOM 195050 (accession no. AY605761.1) as outgroups. TA-1 ITS sequence showed high similarity to *T. yunnanense* and *T. asperellum* without forming a distinct clade; CAL and EF-1α sequences exhibited high similarity to, and clustered with, multiple *T. asperellum* strains. Based on morphology and multilocus phylogenetic analysis, TA-1 was identified as *T. asperellum*.

The topology of the phylogenetic tree showed that TA-1 sequences were located within the *Trichoderma* lineage and were clearly separated from the outgroup taxa. Among the individual loci, ITS placed TA-1 within the broader *Trichoderma* clade but provided limited resolution among closely related species, as the TA-1 ITS sequence showed high similarity to both *T. yunnanense* and *T. asperellum* reference sequences. In contrast, the CAL and EF-1α loci provided stronger species-level resolution, with TA-1 clustering more consistently with multiple *T. asperellum* reference strains. Therefore, compared with ITS alone, the combined multi-locus evidence provided more robust support for assigning TA-1 to *T. asperellum*. Based on the phylogenetic topology together with morphological characteristics, strain TA-1 was identified as *T. asperellum*.

### 3.2 Physiological and biochemical characterization of *T. asperellum* TA-1

The biocontrol potential of an antagonist is often correlated with its ability to produce various bioactive compounds and enzymes. Therefore, we assessed several key physiological traits of *T. asperellum* TA-1. Ammonia production is linked to plant growth promotion, while lytic enzymes such as laccase, protease, and amylase can contribute to both direct antagonism and induced systemic resistance.

Our results demonstrated that TA-1 possesses multiple beneficial traits. In the ammonia production assay, the addition of Nessler’s reagent produced a positive colorimetric reaction. Although the final solution appeared greenish, this color was interpreted as the combined visual effect of the yellow to orange-brown Nessler-ammonia complex and the inherent pigmentation/background of the TA-1 culture broth (Figure 2A). When cultured on a guaiacol-supplemented medium to test for laccase activity, a distinct dark brown halo with a reddish periphery formed around the TA-1 colony, indicating the secretion of laccase (Figure 2B). For the protease activity assay, TA-1 created a clear zone of hydrolysis on the skim milk agar, where the opaque, milky-white medium became transparent around the colony, demonstrating the production of extracellular proteases (Figure 2C). Finally, in the amylase activity test, flooding the starch agar plate with iodine solution revealed a clear, unstained halo around the TA-1 colony, which contrasted with the dark blue color of the surrounding medium. This indicated that TA-1 effectively secretes amylase to hydrolyze starch (Figure 2D).

**FIGURE 2:**
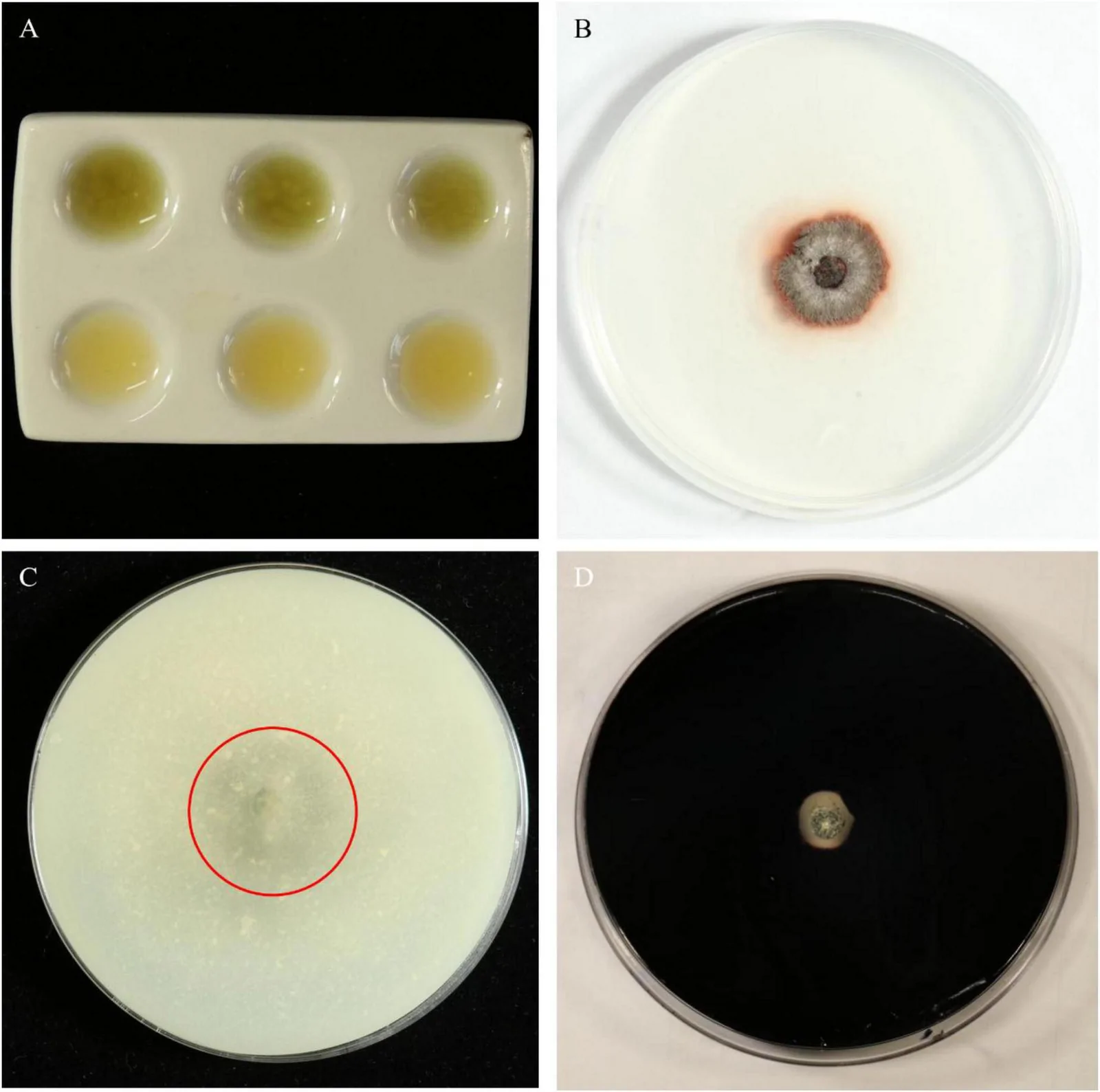
Physiological and biochemical traits of *T. asperellum* TA-1. **(A)** Ammonia production assay showing a positive Nessler colorimetric reaction; the greenish appearance reflects the combined effect of the reaction product and TA-1 culture pigmentation/background; **(B)** laccase activity, indicated by a dark brown halo with a reddish periphery surrounding the colony grown on guaiacol-supplemented medium; **(C)** protease activity, visualized as a clear zone of hydrolysis on skim milk agar; **(D)** amylase activity, demonstrated by a clear, unstained halo on starch agar following iodine staining.

### 3.3 Broad-spectrum antagonistic activity of *T. asperellum* TA-1 against phytopathogenic fungi

The *in vitro* antagonistic potential of *T. asperellum* TA-1 was evaluated against seven phytopathogenic fungi. TA-1 inhibited all tested pathogens (Figures 3A–F and Table 1).

**FIGURE 3:**
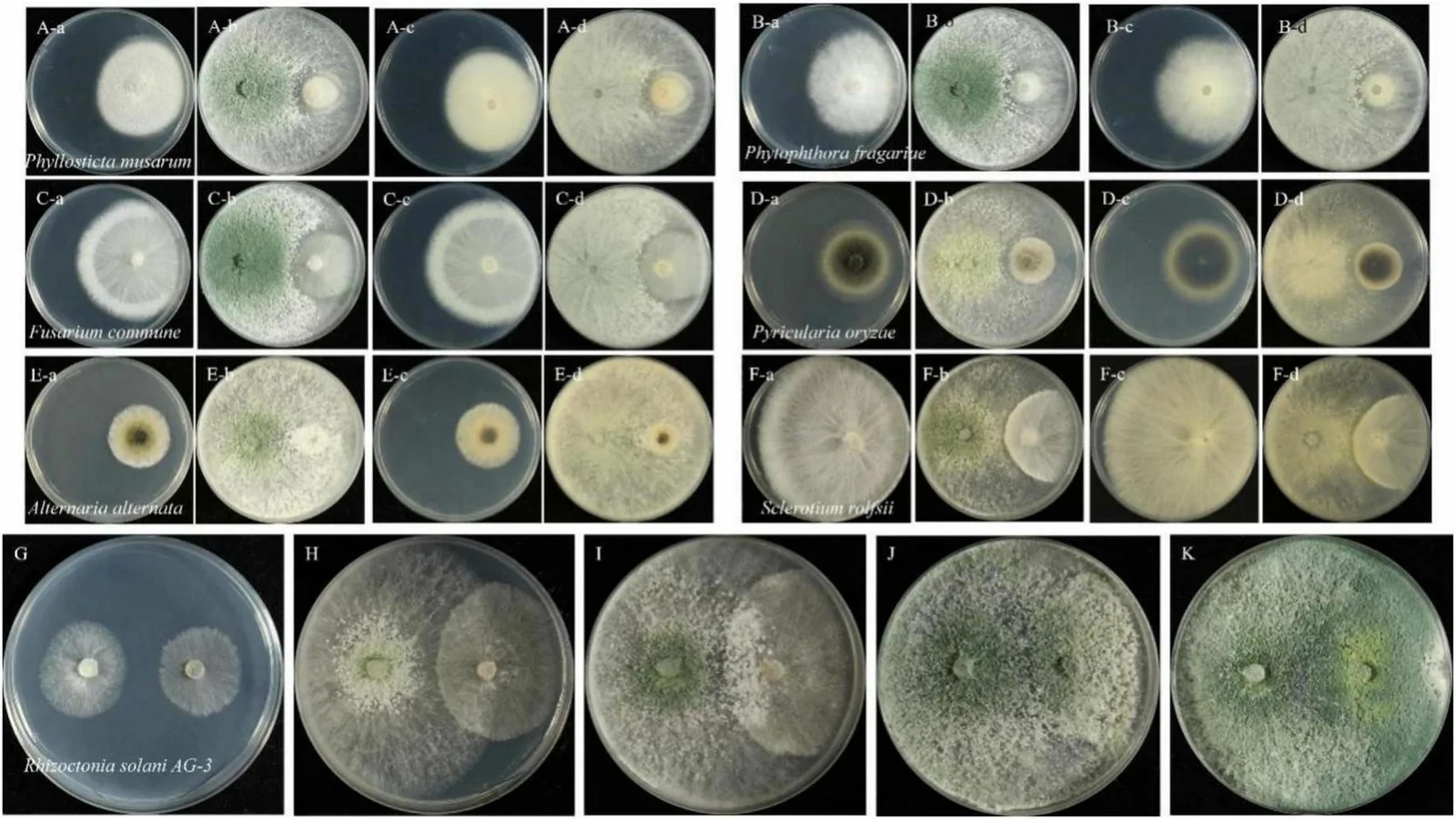
Broad-spectrum antagonistic activity of *T. asperellum* TA-1 against phytopathogenic fungi and its mycoparasitic interaction with *R. solani* AG-3. **(A)** Inhibition of *Phyllosticta musarum*: from left to right, frontal view of *P. musarum* alone; frontal view of *P. musarum* confronted with *T. asperellum* TA-1; reverse view of *P. musarum* alone; reverse view of *P. musarum* confronted with *T. asperellum* TA-1. **(B)** Inhibition of *Phytophthora fragariae*: from left to right, frontal view of *P. fragariae* alone; frontal view of *P. fragariae* confronted with *T. asperellum* TA-1; reverse view of *P. fragariae* alone; reverse view of *P. fragariae* confronted with *T. asperellum* TA-1. **(C)** Inhibition of *Fusarium commune*: from left to right, frontal view of *F. commune* alone; frontal view of *F. commune* confronted with *T. asperellum* TA-1; reverse view of *F. commune* alone; reverse view of *F. commune* confronted with *T. asperellum* TA-1. **(D)** Inhibition of *Pyricularia oryzae*: from left to right, frontal view of *P. oryzae* alone; frontal view of *P. oryzae* confronted with *T. asperellum* TA-1; reverse view of *P. oryzae* alone; reverse view of *P. oryzae* confronted with *T. asperellum* TA-1. **(E)** Inhibition of *Alternaria alternata*: from left to right, frontal view of *A. alternata* alone; frontal view of *A. alternata* confronted with *T. asperellum* TA-1; reverse view of *A. alternata* alone; reverse view of *A. alternata* confronted with *T. asperellum* TA-1. **(F)** Inhibition of *Sclerotium rolfsii*: from left to right, frontal view of *S. rolfsii* alone; frontal view of *S. rolfsii* confronted with *T. asperellum* TA-1; reverse view of *S. rolfsii* alone; reverse view of *S. rolfsii* confronted with *T. asperellum* TA-1. **(G–K)** Confrontation assay between TA-1 and *R. solani* AG-3 over 5 days (1–5 days post-inoculation), showing progressive encroachment, mycoparasitism, and overgrowth by TA-1.

**TABLE 1:** *In vitro* antagonistic activity of *T. asperellum* TA-1 against seven plant pathogenic fungi.

Bioagent	Phytopathogenic fungi						
	*P. musarum* PIRG (%)	*P. fragariae* PIRG (%)	*F. commune* PIRG (%)	*P. oryzae* PIRG (%)	*A. alternate* PIRG (%)	*S. rolfsii* PIRG (%)	*R. solani* AG-3 PIRG (%)
TA-1	78.27 ± 6.23a	76.31 ± 1.88ab	76.10 ± 9.50ab	64.83 ± 8.28c	64.50 ± 0.76c	80.18 ± 1.01a	68.06 ± 4.42bc

PIRG values are presented as mean ± SD (*n* = 3). Different lowercase letters indicate significant differences among pathogens according to one-way ANOVA followed by Tukey’s HSD test (*P* < 0.05).

Percentage inhibition of radial growth values ranged from 64.50 ± 0.76% against *Alternaria alternata* to 80.18 ± 1.01% against *Sclerotium rolfsii*. The inhibition values for *Phyllosticta musarum*, *Phytophthora fragariae*, *Fusarium commune*, *Pyricularia oryzae*, and *R. solani* AG-3 were 78.27 ± 6.23%, 76.31 ± 1.88%, 76.10 ± 9.50%, 64.83 ± 8.28%, and 68.06 ± 4.42%, respectively (Table 1).

In addition to radial-growth inhibition, TA-1 overgrew the colonies of most tested pathogens. Although TA-1 significantly restricted *S. rolfsii*, complete overgrowth was not observed during the assay period. Because no specific defense mechanism of *S. rolfsii* was examined in this study, this difference is reported as an observational phenotype rather than attributed to a particular metabolite or structural feature. The interaction between TA-1 and *R. solani* AG-3 was monitored over 5 days. Following hyphal contact, TA-1 progressively overgrew the AG-3 colony and subsequently formed green conidia on the pathogen mycelium (Figures 3G–K).

### 3.4 Biocontrol efficacy of *T. asperellum* TA-1 against tobacco target spot under greenhouse conditions

At 72 h post-inoculation (hpi), typical disease symptoms of target spot developed in all experimental groups, but the severity of the disease varied significantly among the treatments.

In the control group (inoculated with *R. solani* AG-3 only), tobacco leaves exhibited large, characteristic dark brown, circular lesions, often surrounded by a distinct yellow halo (Figures 4A–C). The average lesion diameter in this group reached 13.81 mm.

**FIGURE 4:**
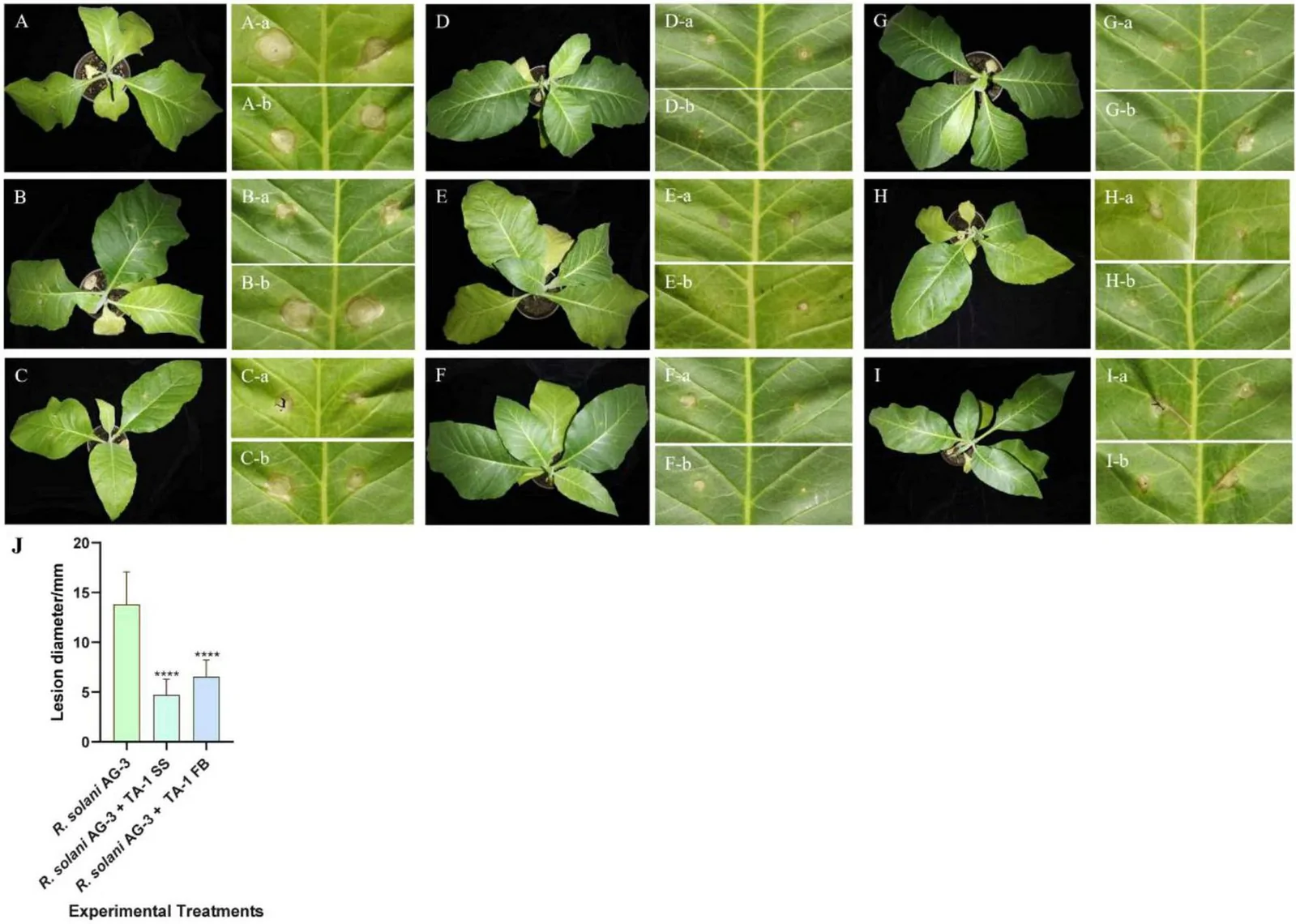
Protective efficacy of *T. asperellum* TA-1 treatments against tobacco target spot caused by *R. solani* AG-3 under greenhouse conditions. **(A–C)** Control group inoculated with *R. solani* AG-3 only, showing typical dark brown, circular lesions with yellow halos; **(D–F)** Tobacco plants treated with TA-1 spore suspension prior to *R. solani* AG-3 inoculation, exhibiting markedly reduced lesion size; **(G–I)** Tobacco plants treated with TA-1 cell-free culture filtrate prior to *R. solani* AG-3 inoculation, showing moderate reduction in lesion size compared to the control. **(J)** Quantitative analysis of the average lesion diameters among different treatments at 72 h post-inoculation. Error bars represent the standard deviation (SD) of the means. Asterisks denote statistically significant differences between the TA-1 treated groups (SS and FB) and the *R. solani* AG-3 control group according to Tukey’s HSD test (*P* < 0.0001).

Plants treated with the TA-1 spore suspension showed smaller lesions, with a mean lesion diameter of 4.72 mm (Figures 4D–F), whereas those treated with the cell-free fermentation filtrate had a mean lesion diameter of 6.55 mm (Figures 4G–I). Both treatments significantly reduced lesion diameter compared with the pathogen control (Figure 4J). Based on lesion diameter, the disease control efficacy (DCE) was 65.82% for the spore suspension and 52.57% for the fermentation filtrate.

### 3.5 Mycoparasitic mechanism of *T. asperellum* TA-1 against *R. solani* AG-3

To elucidate the direct antagonistic mechanism of *T. asperellum* TA-1 against *R. solani* AG-3, the interactions between their hyphae and the effect on pathogen sclerotia were examined using light microscopy and SEM.

Light microscopy observations of the interaction zone revealed that the hyphae of TA-1 actively coiled around and attached to the hyphae of AG-3 (Figures 5A,B). Further examination by SEM provided detailed evidence of this mycoparasitic process. In contrast to the smooth and turgid hyphae observed in the control group (Figure 5C), pathogen hyphae parasitized by TA-1 exhibited severe structural damage, including surface erosion, shriveling, and collapse (Figure 5D).

**FIGURE 5:**
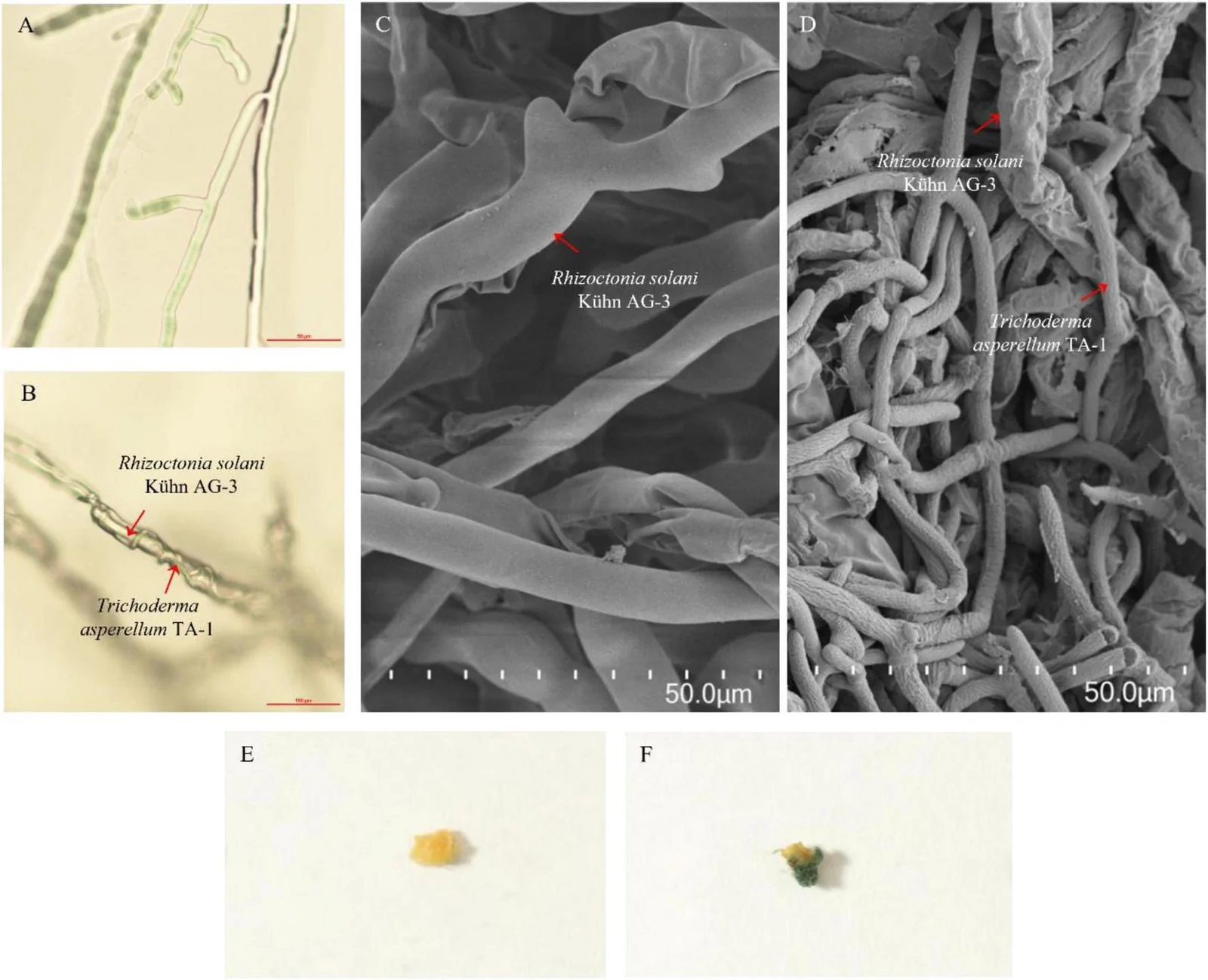
Mycoparasitic interaction of *T. asperellum* TA-1 with *R. solani* AG-3 observed by light microscopy and SEM. **(A,B)** Light microscopy images showing TA-1 hyphae coiling around and attaching to AG-3 hyphae; **(C,D)** SEM images showing hyphal interactions between TA-1 and *R. solani* after co-culture, in which TA-1 hyphae attached to and coiled around *R. solani* hyphae. **(E,F)** Sclerotial colonization assay showing the attachment and colonization of *R. solani* sclerotia by TA-1.

TA-1 also colonized the sclerotial surface of *R. solani* AG-3. Following application of TA-1 spores, spore germination and the development of a proliferating mycelial network were observed on the sclerotial surface (Figures 5E,F), demonstrating the ability of TA-1 to attach to and colonize AG-3 sclerotia under the assay conditions.

### 3.6 Optimization of solid-state fermentation conditions for TA-1 sporulation

Preliminary screening indicated that substrate type, carbon source, nitrogen source, and fermentation time all had significant effects on TA-1 sporulation (Figures 6A–E). Under a moisture content equal to one-fold substrate weight and 1% glucose addition, wheat bran supported the highest spore yield (3.01 × 10^9^ CFU/g dry substrate), whereas soybean meal resulted in the lowest (0.57 × 10^9^ CFU/g dry substrate). Considering its low cost and high yield, wheat bran was selected as the basal substrate for further experiments.

**FIGURE 6:**
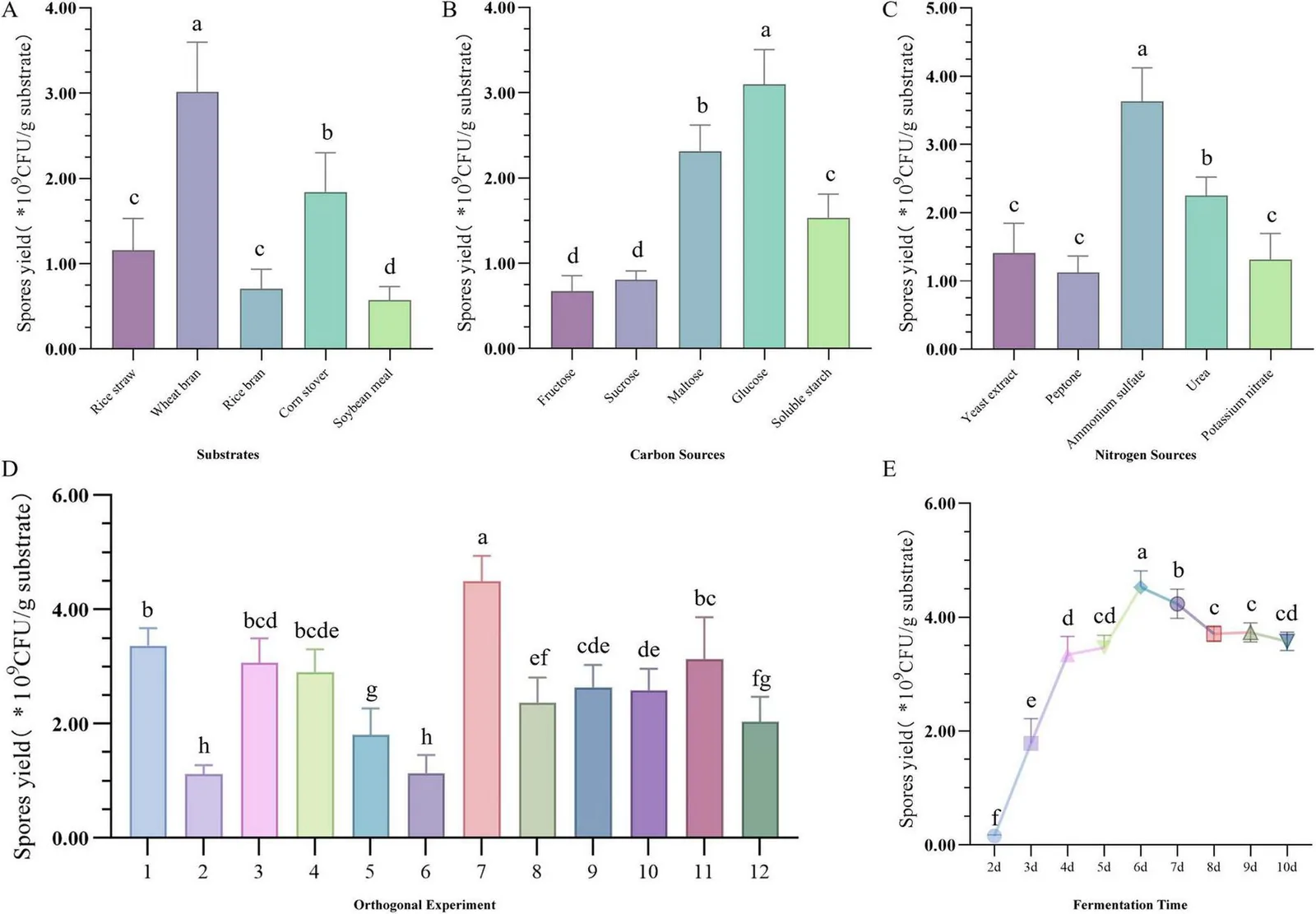
Optimization of solid-state fermentation conditions for spore production by *T. asperellum* TA-1. **(A)** Effect of different substrates on spore yield under initial moisture content of 1× and 1% glucose, showing highest yield with wheat bran (3.01 × 10^9^CFU/g dry substrate) and lowest with soybean meal (0.57 × 10^9^CFU/g dry substrate); **(B)** effect of different carbon sources (1% w/w) on spore yield, with maximum production observed with glucose (3.10 × 10^9^CFU/g dry substrate) and minimum with fructose (0.67 × 10^9^CFU/g dry substrate); **(C)** effect of different nitrogen sources (1% w/w) under fixed carbon source conditions, showing highest yield with ammonium sulfate (3.63 × 10^9^CFU/g dry substrate) and lowest with peptone (1.12 × 10^9^CFU/g dry substrate); **(D)** results of orthogonal experiment on substrate moisture, carbon source concentration, and nitrogen source concentration, identifying optimal formulation (1× moisture, 0.5% glucose, 1.5% ammonium sulfate) yielding 4.74 × 10^9^CFU/g dry substrate; **(E)** effect of fermentation time on spore yield, showing an increasing trend from day 2 to day 6 (peak at 4.74 × 10^9^CFU/g dry substrate) followed by decline from day 7, with significant reduction by day 10. Data are presented as mean ± SD (*n* = 3). Different lowercase letters within each panel indicate statistically significant differences among treatments, as determined by one-way ANOVA followed by Tukey’s HSD test (*P* < 0.05).

When glucose was used as the carbon source at a 1% supplementation level, the spore yield reached 3.10 × 10^9^ CFU/g dry substrate, which was higher than that obtained with fructose (0.67 × 10^9^ CFU/g dry substrate). For nitrogen sources, ammonium sulfate (3.63 × 10^9^ CFU/g dry substrate) significantly outperformed peptone (1.12 × 10^9^ CFU/g dry substrate). Consequently, glucose and ammonium sulfate were selected as the carbon and nitrogen sources, respectively, for orthogonal design experiments.

Orthogonal optimization revealed that the optimal combination was 1-fold moisture content (Table 2), 0.5% glucose, and 1.5% ammonium sulfate, yielding 4.74 × 10^9^ CFU/g dry substrate, which was significantly higher than other tested combinations. The fermentation time experiment showed an increasing sporulation trend from day 2 to day 6, peaking at day 6 (4.74 × 10^9^ CFU/g dry substrate), followed by a gradual decline to 3.57 × 10^9^ CFU/g dry substrate by day 10. Thus, the optimal solid-state fermentation conditions for TA-1 were determined to be: wheat bran substrate, 1-fold moisture content, 0.5% glucose, 1.5% ammonium sulfate, and 6 days of fermentation.

**TABLE 2:** Orthogonal experimental design and results of solid-state fermentation medium optimization for *T. asperellum* TA-1.

Experimental orthogonal and results of TA-1				
Group	A Water content (×)	B Carbon source (%)	C Nitrogen source (%)	Spore number × 10^9^ CFU/g dry substrate
1	1.5	0.5	1.5	3.36 ± 0.30b
2	2	1.5	1	1.12 ± 0.16g
3	1.5	1.5	1.5	3.07 ± 0.43bc
4	2	1	1.5	2.9 ± 0.40bcd
5	1	1.5	1	1.8 ± 0.46f
6	1.5	1.5	0.5	1.13 ± 0.32g
7	1	0.5	1.5	4.74 ± 0.44a
8	1	1	1.5	2.37 ± 0.44de
9	1.5	0.5	0.5	2.63 ± 0.40cd
10	1	1	0.5	2.58 ± 0.38cd
11	2	1	0.5	3.13 ± 0.72b
12	2	0.5	1	2.03 ± 0.44ef

Values represent mean ± standard deviation (*n* = 3). Different lowercase letters (a, b, c, d, e, f, g) within the same column indicate statistically significant differences among treatments according to one-way ANOVA followed by Tukey’s HSD test at *P* < 0.05. Water content (×) refers to the ratio of added water to the substrate; carbon source (%) was provided as glucose; nitrogen source (%) was provided as ammonium sulfate.

### 3.7 Transcriptomic response of *T. asperellum* TA-1 during mycoparasitic interaction

A total of nine transcriptomic libraries were constructed, comprising three biological replicates each for TA-1 cultured alone, *R. solani* AG-3 cultured alone, and the TA-1–*R. solani* AG-3 co-culture interaction group. Across the nine libraries, a total of 36.03 Gb of raw sequencing data was generated, yielding 34.98 Gb of high-quality clean data after removal of low-quality reads, with an average GC content of 52.17%. For the transcriptomic analysis of TA-1, clean reads from the six TA-1-containing libraries, including the TA-1 monoculture and TA-1–*R. solani* AG-3 co-culture groups, were aligned to the *T. asperellum* FT101 reference genome (GCA_020647865.1). The overall alignment rates ranged from 87.39% to 96.19%, with an average mapping rate of 93.01%.

Comparative analysis of differentially expressed genes (DEGs) in *T. asperellum* TA-1 between the control and TA-1–*R. solani* AG-3 co-culture groups identified 3,681 DEGs during the antagonistic interaction, including 2,505 upregulated and 1,176 downregulated genes (Figure 7A).

**FIGURE 7:**
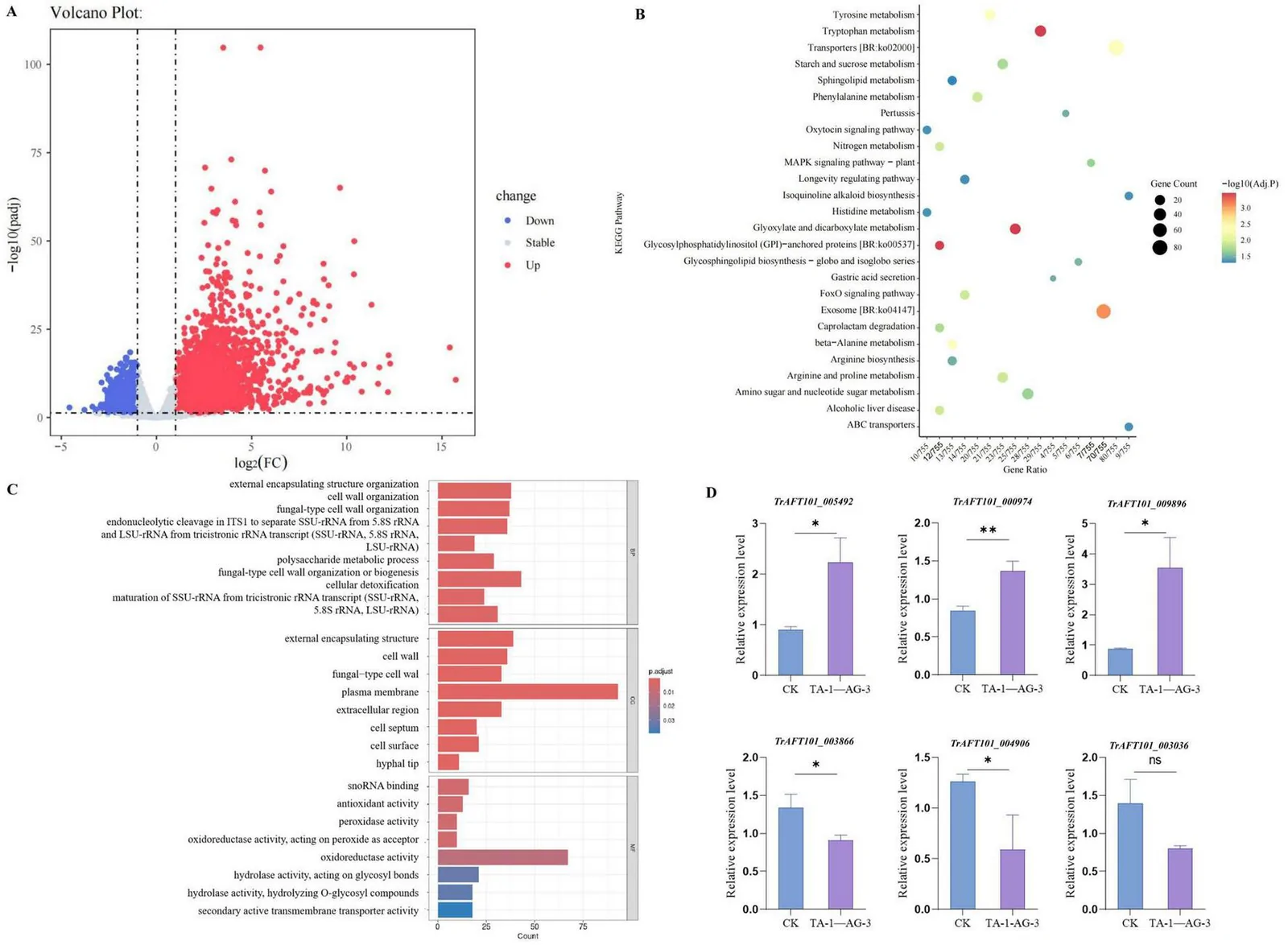
Transcriptomic and RT-qPCR analyses of *T. asperellum* TA-1 during antagonistic interaction with *R. solani* AG-3. **(A)** Volcano plot of differentially expressed genes (DEGs) between TA-1 control and TA-1–AG-3 co-culture groups, indicating 2,505 upregulated and 1,176 downregulated genes; **(B)** KEGG pathway enrichment analysis of DEGs, with significant representation in transporters (ko02000), exosome-related processes (ko04147), glyoxylate and dicarboxylate metabolism (ko00630), and tryptophan metabolism (ko00380); **(C)** GO enrichment analysis of DEGs, classified into Biological Process, Cellular Component, and Molecular Function categories; **(D)** RT-qPCR validation of six selected DEGs (*TrAFT101_005492*, *TrAFT101_000974*, *TrAFT101_009896*, *TrAFT101_003866*, *TrAFT101_004906*, *TrAFT101_003036*), showing concordant expression trends with RNA-seq results. Data are presented as mean ± SD (*n* = 3). In panel **D**, * and ** indicate statistically significant differences according to one-way ANOVA followed by Tukey’s HSD test (*P < 0.05, **P < 0.01); ns indicates no significant difference.

KEGG enrichment analysis identified 494 DEGs associated with pathways including transporters (ko02000), exosome-related processes (ko04147), glyoxylate and dicarboxylate metabolism (ko00630), and tryptophan metabolism (ko00380) (Figure 7B). These enrichments indicate biological processes potentially associated with the TA-1–*R. solani* AG-3 interaction but do not, by themselves, establish causal roles for individual genes or pathways.

GO enrichment analysis revealed that 1,985 DEGs were significantly enriched across the three major GO categories (Figure 7C). In the Biological Process category, highly represented terms included developmental process, amino acid metabolic process, and anatomical structure development. In the Cellular Component category, DEGs were enriched in the nucleolus and site of polarized growth. In the Molecular Function category, oxidoreductase activity was among the most enriched terms. Further analysis showed that upregulated genes were mainly associated with site of polarized growth, anatomical structure development, and developmental process, whereas downregulated genes were primarily enriched in nucleolus-related functions.

Six candidate DEGs were selected for RT-qPCR validation, including three upregulated genes, *TrAFT101_005492* (CFEM domain protein; log_2_FC = 4.99), *TrAFT101_000974* (lipoxygenase; log_2_FC = 4.73), and *TrAFT101_009896* (hydantoinase B/oxoprolinase; log_2_FC = 4.18), and three downregulated genes, *TrAFT101_004906* (histidine acid phosphatase; log_2_FC = −1.84), *TrAFT101_003036* (GPR1/FUN34/yaaH family protein; log_2_FC = −2.08), and *TrAFT101_003866* (xylose isomerase-like TIM barrel protein; log_2_FC = −2.18). The RT-qPCR results showed directional agreement with the RNA-seq data for all six genes (Figure 7D).

Notably, the strong upregulation of *TrAFT101_005492* and *TrAFT101_000974* occurred concurrently with the hyphal attachment, coiling, and structural disruption of *R. solani* AG-3 observed microscopically (Figures 5A–D). These transcriptional responses were consistent with the observed mycoparasitic phenotype and suggest that these genes may be associated with the interaction between TA-1 and *R. solani* AG-3, although their specific functional roles require further validation.

## 4 Discussion

Soil-borne fungal diseases pose a persistent threat to agricultural production due to their high pathogenicity and long-term survival in natural environments. *R. solani*, a broad-spectrum pathogen, can cause a wide range of economically important diseases, including tobacco target spot and rice sheath blight (Gonzalez et al., 2011). Its capability of forming sclerotia allows long-term survival in soil, significantly increasing the risk of disease recurrence (Tsror, 2010). Conventional control strategies rely heavily on chemical fungicides, which, although effective in the short term, have been associated with environmental pollution, soil microbiome disruption, and the emergence of fungicide resistance (Islam et al., 2024). In recent years, biological control using antagonistic microorganisms has emerged as an environmentally friendly approach for plant disease management, among which fungi of the genus *Trichoderma* have attracted considerable attention due to their multi-mechanistic antagonistic activities and wide ecological adaptability (Harman et al., 2004; Harman, 2006).

In this study, TA-1 was isolated from rhizosphere soil in a tobacco–rice rotation system in Chenzhou, Hunan Province, China, and identified as *T. asperellum* based on morphological characteristics and multilocus analysis of the ITS, CAL, and EF-1α regions. Dual-culture assays showed that TA-1 inhibited all seven tested phytopathogenic fungi, with PIRG values ranging from 64.50 ± 0.76% to 80.18 ± 1.01%. Against the focal pathogen, *R. solani* AG-3, the PIRG was 68.06 ± 4.42%. Microscopic observations revealed hyphal attachment and coiling during the TA-1–AG-3 interaction, while SEM showed pronounced structural alterations in AG-3 hyphae, including surface erosion, shriveling, and collapse. In addition, TA-1 spores germinated and developed an extensive mycelial network on the surface of AG-3 sclerotia. Together, these observations support mycoparasitism as an important component of TA-1 antagonism, although they do not establish the specific contributions of individual enzymes, metabolites, or genes.

Qualitative functional screening further showed ammonia production and positive laccase, protease, and amylase activities in TA-1. These traits are consistent with potential roles in competition and mycoparasitic interactions. Ammonia production has previously been associated with direct antifungal effects and modulation of plant defense responses (Norred, 1982; Nautiyal, 1999), whereas extracellular hydrolytic enzymes such as proteases may contribute to the degradation of pathogen-associated macromolecules during antagonistic interactions (Harman et al., 2004). Laccase activity may also participate in oxidative and detoxification processes associated with fungal interactions (Mayer and Staples, 2002; Pourcel et al., 2005). However, the assays used in the present study were qualitative plate- or color-based screening methods and were not calibrated for enzyme activity units or halo-diameter measurements. Therefore, these observations should be interpreted as qualitative evidence of functional traits rather than quantitative measures of their contribution to disease suppression. Likewise, visible colonization of AG-3 sclerotia does not demonstrate loss of sclerotial viability. Quantitative enzyme assays, sclerotial viability tests, and targeted functional analyses will be required to determine the direct contribution of these traits to antagonism.

At the molecular level, transcriptomic analysis revealed substantial transcriptional reprogramming of TA-1 during interaction with AG-3, with 3,681 DEGs identified, including 2,505 upregulated and 1,176 downregulated genes. KEGG and GO enrichment analyses highlighted candidate processes associated with transport, metabolism, oxidoreductase activity, polarized growth, and development. Notably, *TrAFT101_005492*, encoding a CFEM domain protein, and *TrAFT101_000974*, encoding a lipoxygenase, were strongly upregulated, with log_2_FC values of 4.99 and 4.73, respectively. Their transcriptional induction occurred concurrently with the observed hyphal attachment, coiling, and structural disruption of AG-3, suggesting a potential association with the mycoparasitic interaction. CFEM domain proteins have been implicated in fungal cell-surface processes and host interactions (Chen et al., 2021), whereas lipoxygenases participate in oxylipin metabolism and oxidative-response pathways (Porta and Rocha-Sosa, 2002). However, the present transcriptomic data provide associative rather than direct functional evidence, and the specific roles of these candidate genes in TA-1 mycoparasitism remain to be experimentally validated.

RT-qPCR analysis showed directional agreement with the RNA-seq expression patterns of the selected candidate genes. Actin was used as the RT-qPCR reference gene based on its previous application in *Trichoderma* gene-expression studies (Díaz-Gutiérrez et al., 2021). Nevertheless, reference-gene suitability can be condition dependent, and future studies should evaluate multiple candidate reference genes, such as Tubulin and EF-1α, to further assess normalization stability under dynamic TA-1–*R. solani* AG-3 interaction conditions.

For potential application, solid-state fermentation conditions were established using wheat bran as the substrate, a water-to-substrate ratio of 1:1, 0.5% glucose, and 1.5% ammonium sulfate. Under these conditions, the maximum spore yield reached 4.74 × 10^9^ CFU/g dry substrate on day 6. These results provide laboratory-scale parameters for the mass production of TA-1. Greenhouse assays further demonstrated that TA-1 significantly reduced tobacco target spot severity, with disease control efficacy values of 65.82% for the spore suspension and 52.57% for the cell-free fermentation filtrate. However, greenhouse conditions do not fully represent the complexity of agricultural environments, where temperature, humidity, soil properties, microbial communities, and cropping practices may influence strain survival and efficacy. Field validation is therefore part of the continuing evaluation of TA-1, and further multi-location and multi-season trials will be important for assessing the consistency, persistence, and adaptability of its biocontrol performance under agricultural conditions.

Collectively, the present results demonstrate that *T. asperellum* TA-1 possesses broad *in vitro* antagonistic activity, exhibits clear morphological features associated with mycoparasitic interaction with *R. solani* AG-3, and provides significant disease suppression under greenhouse conditions. Transcriptomic analysis further identified candidate genes and biological processes potentially associated with this interaction, while solid-state fermentation optimization established practical parameters for spore production. These findings support further development of TA-1 as a biocontrol candidate for tobacco target spot while also highlighting the need for additional functional validation of candidate mechanisms and broader field evaluation.

## 5 Conclusion

In this study, a fungal antagonist, identified as *T. asperellum* and designated as strain TA-1, was successfully isolated from soil. This strain demonstrated potent, broad-spectrum antagonistic activity against a range of tested phytopathogens, with PIRG values ranging from 64.50 ± 0.76% to 80.18 ± 1.01%. Against the focal pathogen, *R. solani* AG-3, the PIRG was 68.06 ± 4.42%. TA-1 also showed qualitative evidence of ammonia production and laccase, protease, and amylase activities.

Our investigation focused specifically on the antagonism of TA-1 against *R. solani* AG-3, the causal agent of tobacco target spot. TA-1 exhibited strong mycoparasitic characteristics, including hyphal attachment and coiling, pronounced structural damage to pathogen hyphae, and colonization of the AG-3 sclerotial surface. In greenhouse trials, the TA-1 spore suspension and fermentation filtrate achieved DCE values of 65.82% and 52.57%, respectively, demonstrating significant suppression of tobacco target spot.

Furthermore, transcriptome analysis provided preliminary insights into candidate genes associated with the TA-1–AG-3 interaction. The solid-state fermentation process for TA-1 was also optimized, with a final spore yield of 4.74 × 10^9^ CFU/g dry substrate under the optimized conditions. Collectively, these findings identify *T. asperellum* TA-1 as a promising biocontrol agent for tobacco target spot and provide a foundation for further mechanistic validation and practical application.

## Data Availability

The raw transcriptome sequence data have been deposited in the National Center for Biotechnology Information (NCBI) Sequence Read Archive (SRA) under BioProject accession PRJNA1346907 and accession numbers SRR38967827–SRR38967835.
