# Supplementary material for: Biocontrol efficacy of Trichoderma asperellum TA-1 against Rhizoctonia solani AG-3 and its underlying mechanisms
Source: Front Microbiol. 2026 Oct 13;17:1899737. doi: 10.3389/fmicb.2026.1899737 (PMC13710277; doi:10.3389/fmicb.2026.1899737)
Supplement: Supplementary file 1 [file Table_1.docx]

Table S1. Primer sequences used for PCR amplification of *T. asperellum* TA‑1.

| Primers | Sequence (5’–3’) | Product size (bp) | Reference |
| --- | --- | --- | --- |
| ITS1 | TCCGTAGGTGAACCTGCGG | 606 | (Qin *et al.*,2024)^[15]^ |
| ITS4 | TCCTCCGCTTATTGATATGC |  |  |
| CAL-228F | GAGTTCAAGGAGGCCTTCTCCC | 502 | (Matar *et al.*, 2022)^[14]^ |
| CAL-737R | CATCTTTCTGGCCATCATGG |  |  |
| EF1-728F | CATCGAGAAGTTCGAGAAGG | 304 | (Matar *et al.*, 2022)^[14]^ |
| EF1-986R | TACTTGAAGGAACCCTTACC |  |  |
