# Supplementary material for: Biocontrol efficacy of Trichoderma asperellum TA-1 against Rhizoctonia solani AG-3 and its underlying mechanisms
Source: Front Microbiol. 2026 Oct 13;17:1899737. doi: 10.3389/fmicb.2026.1899737 (PMC13710277; doi:10.3389/fmicb.2026.1899737)
Supplement: Supplementary file 2 [file Table_2.docx]

Table S2. Primer sequences used for RT‑qPCR analysis of selected genes.

| Primers | Sequence (5’–3’) | Primers | Sequence (5’–3’) |
| --- | --- | --- | --- |
| TrAFT101_0098-F | AGGAAAAGGACGCAATAGGG | TrAFT101_0098-R | AGACTGAATCACAACGCTCTC |
| TrAFT101_000974-F | AGTCCCCTGTACCTCTCAAG | TrAFT101_000974-R | TCATCTCCACCTTGTTCACTG |
| TrAFT101_005492-F | GAAGTAATCCATGGCCCTAAGG | TrAFT101_005492-R | ATCTATCCCCGCATTGTGAAG |
| TrAFT101_003866-F | TGCGTACAAGATGAAACCTCC | TrAFT101_003866-R | GGCAGATACCCTCCGTTAAAG |
| TrAFT101_003036-F | TTTGGGTACTGTTGGTCCTG | TrAFT101_003036-R | GGACAAGGAACATGGCAAAAG |
| TrAFT101_004906-F | CAATCAAGCTCTGCCTTTCAAG | TrAFT101_004906-R | GAACCGAAGCCAAAATCCAC |
| Actin-F | GCTGAGCGAGAAATCGTTCGT | Actin-R | AGCAGTCTGGATCTCCTGCTC |
